# COVID‐19: Face masks and human‐to‐human transmission

**DOI:** 10.1111/irv.12740

**Published:** 2020-04-05

**Authors:** Xiaopeng Liu, Sisen Zhang

**Affiliations:** ^1^ Henan University of Chinese Medicine Zhengzhou, Henan China; ^2^ Department of Emergency People's Hospital of Henan University of Chinese Medicine Zhengzhou, Henan China; ^3^ Zhengzhou People's Hospital Southern Medical University Zhengzhou, Henan China

**Keywords:** COVID‐19, face masks, infectious disease

Dear Editor,

In December 2019, transmission of the novel coronavirus (SARS‐CoV‐2) that causes coronavirus disease 2019 (COVID‐19) occurred in Wuhan, China.^1^ And later, the virus began to be transmitted from person to person.^2^ Face masks are a type of personal protective equipment used to prevent the spread of respiratory infections, and it may be effective at helping prevent transmission of respiratory viruses and bacteria.^3^ Here, we share a case of face masks are being used to prevent the transmission of COVID‐19 infection.

We report a typical case of cluster outbreak caused by public transportation exposure; during the outbreak of COVID‐19 epidemic, one patient from Chongqing, China, did not wear a face mask in the first vehicle while wore a face mask in the second vehicle he took. One male patient with COVID‐19 found himself coughing. Unaware of the fact that he might have been infected with COVID‐19 and in a hurry, he did not manage to get a face mask before he took the coach bus from the city back to his county. Many passengers did not wear face masks on the same coach bus. The duration of this bus was 2 hours and 10 minutes, and there were 39 other passengers on the same coach bus. According to epidemiological survey, 5 other passengers on the same coach bus were infected. Upon arrival in the county, this male patient bought a face mask and took a minibus to his final destination wearing the mask. The duration of minibus was 50 minutes, and there were 14 other passengers on the same minibus. The Center for Disease Control and Prevention conducted an epidemiological investigation and close contact tracing management. The passengers on the minibus were screened and treated as suspected cases. A 14‐day medical observation period was conducted. During the observation period, passengers were taken temperature twice a day and and were asked whether they had respiratory symptoms such as fever and dry cough, or digestive symptoms such as diarrhea. All the passengers did not have fever, cough, or other abnormal symptoms, and two quantitative reverse transcriptase–polymerase chain reaction (qRT‐PCR) test results were negative. No passengers were infected COVID‐19 in the same minibus.

During the outbreak of COVID‐19, one patient from Chongqing, China, has transmitted the COVID‐19 to 5 people in one vehicle when he did not wear a face mask while no one was infected later in the second vehicle he took when he wore a face mask, indicating the importance of wearing face masks for everyone in a closed space. Previous news reported that The Australian Federal Government has released 500 000 face masks to general practitioners and other health workers across the country to protect them from infection by COVID‐19.^4^ Wearing face masks protects yourself and others. Use of face masks is therefore likely to play a vital role in mitigating disease spread.^5^

When taking long‐distance public transportation, one should first evaluate his/her own health conditions. Avoid the trip if any symptoms like fever or coughing are present and go to fever clinics as soon as possible, wearing a face mask. Wear a face mask during the entire trip. It is advised that you bring 1‐2 extra face masks and wear a new face mask immediately if the old one is deformed or contaminated. Face masks are recommended for diseases transmitted through droplets and respirators for respiratory aerosols and may prevent infection in public settings.^6^ The potential of face masks to reduce the spread of respiratory infections and could be useful.^7^ In the study^8^ of attitudes of influenza‐vaccinated healthcare workers toward masks, 65.7% of the participants agreed with infection control recommendation “wearing a mask” to prevent influenza transmission. Due to the lack of research on face masks, further research should focus on assessing the efficacy of face masks against COVID‐19, investigating reuse of face masks and assessing compliance.

## CONFLICT OF INTEREST

The authors declare that they have no competing interests.

## Author Contribution

**Xiaopeng Liu:** Writing‐original draft (equal); Writing‐review & editing (equal). **Sisen Zhang:** Writing‐original draft (equal); Writing‐review & editing (equal).
